# The Role of Granulocyte Colony Stimulating Factor in Mitigating the Risk of Recurrent Miscarriages: A Comprehensive Review and Meta-Analysis

**DOI:** 10.1007/s43032-024-01736-z

**Published:** 2024-12-02

**Authors:** Biyun Zhang, Jianying Xu, Xiangcai Wei, Xingming Zhong

**Affiliations:** 1 Reproductive Immunization Center, Guangdong Provincial Reproductive Science Institute (Guangdong Provincial Fertility Hospital), NHC Key Laboratory of Male Reproduction and Genetics, Guangzhou, Guangdong 510660 China; 2 Center of Reproductive Medicine, Zhuhai Center for Maternal and Child Health Care, Zhuhai, Guangdong 519000 China; 3 Department of Reproductive Immunity, Guangdong Women and Children Hospital, Guangzhou Medical University, No. 13, Guangyuan West Road, Yuexiu District, Guangzhou, Guangdong 510060 China

**Keywords:** G-CSF, Treatment, Miscarriage, Clinical, Evidence

## Abstract

Granulocyte colony-stimulating factor (G-CSF), a pivotal hematopoietic cytokine, has been noted for its potential to bolster embryo implantation and augment endometrial receptivity. The present meta-analysis endeavors to evaluate the therapeutic efficacy of G-CSF in mitigating the incidence of recurrent miscarriages, thereby enriching the clinical evidence supporting its use in treatment protocols. Our exhaustive literature search, concluded on August 25, 2024, spanned across various databases including PubMed, Medline, Cochrane Library, Web of Science, ClinicalTrials, China National Knowledge Infrastructure (CNKI), Weipu, and Wanfang, to identify and analyze randomized controlled trials (RCTs) that assessed the impact of G-CSF on recurrent miscarriage. Our review incorporated 7 RCTs. The application of G-CSF was linked to a marked reduction in the rate of miscarriage [RR = 0.48, 95% CI (0.27, 0.86), *P* = 0.01]. Subgroup analysis indicated that the intra-uterine infusion of G-CSF was notably effective in diminishing the miscarriage rate (RR = 0.35, 95% CI (0.18, 0.68), *P* = 0.002), while subcutaneous administration did not exhibit a significant impact (RR = 0.55, 95% CI (0.26, 1.20), *P* = 0.13). Moreover, the administration of G-CSF during the ovulatory phase was identified as particularly efficacious in reducing the miscarriage rate (RR = 0.33, 95% CI (0.18, 0.63), *P* < 0.001). Intrauterine administration of G-CSF, particularly during the ovulatory phase, is associated with a significant decrease in miscarriage risk and an enhancement in the likelihood of a successful pregnancy outcome in patients with a history of recurrent miscarriages. These findings highlight G-CSF’s promise as a valuable therapeutic intervention in this medical scenario.

## Introduction

Spontaneous abortion is one of the common clinical diseases, with its etiological factors encompassing endocrine disorders, immune system abnormalities, et al. [1]. It is estimated that approximately 25% of women will experience a miscarriage at some point in their lives, with up to 5% having two or more miscarriages [2]. In recent years, influenced by societal changes and increased life pressures, human fertility has been on a declining trend. Consequently, the incidence of infertility, repeated implantation failures, and recurrent miscarriages has been on the rise [3]. Recurrent miscarriage is defined as the occurrence of two or more consecutive spontaneous abortions in a woman of reproductive age with the same sexual partner. It is currently believed that the pathogenesis is associated with genetics, anatomical abnormalities, immune status, endocrine disorders et al. [4, 5]. The prevalence of recurrent miscarriage accounts for 1–5% of all pregnancies, with the risk of recurrence increasing with the number of miscarriages [6]. Recurrent miscarriage has a profound impact on the physical and mental health of women and their fertility, posing a clinical challenge [7]. Therefore, actively seeking effective treatment strategies is of significant importance for the prognosis of patients with recurrent miscarriage.

In recent years, an increasing number of scholars have begun to focus on the role of granulocyte colony-stimulating factor (G-CSF) in reproductive immunology. G-CSF is a specific hematopoietic cytokine primarily produced by bone marrow cells, macrophages, and fibroblasts [8]. Research [9] has shown that G-CSF can induce the proliferation, invasion, and maintenance of trophoblasts during pregnancy. Studies [10, 11] have reported that it improves the endometrial receptivity of patients by promoting endometrial vascular remodeling, embryo adhesion and invasion, and regulating the endometrial immune environment, suggesting potential therapeutic applications in the treatment of recurrent miscarriage. Previous research [12] has indicated that G-CSF can facilitate embryo implantation, thicken the endometrium, and reduce the risk of miscarriage. However, some studies [13, 14] suggest that the application of G-CSF in early pregnancy does not significantly improve the prognosis of patients with recurrent miscarriage. To further explore the clinical efficacy of G-CSF in the treatment of recurrent miscarriage, this study employs a meta-analysis to systematically evaluate the effects of G-CSF therapy on recurrent miscarriage, with the aim of providing more evidence for clinical treatment of recurrent miscarriage.

## Methods

This meta-analysis was conducted according to the Preferred Reporting Items for Systematic reviews and Meta-Analyses (PRISMA) statement [15]. As this study was a meta-analysis, ethical approval and patient informed consent were not required.

### Literature Search

We conducted a comprehensive literature search in databases including PubMed, Medline, Cochrane Library, Web of Science, ClinicalTrials, China National Knowledge Infrastructure (CNKI), Weipu, and Wanfang, to identify randomized controlled trials (RCTs) evaluating the efficacy of G-CSF in the treatment of recurrent miscarriage. The search was performed up to August 25, 2024, covering the inception of the databases. The meta-analysis employed the following search strategy: (“Miscarriage” OR “Abortion” OR “Pregnancy”) AND (“Granulocyte Colony-Stimulating Factor” OR “G-CSF”). The search strategy integrated both MeSH terms and free-text keywords.

### Inclusion and Exclusion Criteria

The criteria for study inclusion in this research were as follows: Inclusion Criteria: (1) Study Design: RCTs; (2) Study Population: Patients with recurrent miscarriage; (3) Intervention: The control group received routine treatment, while the intervention group receives G-CSF therapy in addition to routine care; (4) Outcome measures: Abortion rate and live birth rate. Exclusion criteria: (1) Basic research involving experimental animals; (2) Literature not in Chinese or English; (3) Literature for which the full text was inaccessible (4) Review articles, case reports, or duplicated publications.

### Literature Screening and Data Extraction

Two researchers independently conducted searches in the databases, initially excluding literature that clearly did not meet the inclusion criteria based on the titles and abstracts. Full texts of potentially eligible studies were then reviewed. The two researchers cross-checked the selected literature and any discrepancies were resolved by a third researcher. The final included studies were subjected to data extraction, which included the following items: first author, publication date, sample size, age, intervention measures, duration and frequency of intervention, outcomes and conclusions.

### Assessment of Study Quality

The quality of the included studies was assessed using the risk of bias criteria [16] recommended by the “Cochrane Handbook for Systematic Reviews of Interventions Version 5.1.0” from the Cochrane Collaboration. The evaluation encompassed seven aspects: Generation of random sequences; allocation concealment; blinding of participants and personnel; blinding of outcome assessors; incomplete outcome data; selective reporting; other sources of bias. Each item could be rated as “low risk,” “high risk,” or “unclear risk.”

### Statistical Methods

This study employed the RevMan 5.4 statistical software provided by the Cochrane Collaboration for conducting a meta-analysis. For binary outcome variables, the relative risk (RR) with its 95% confidence interval (CI) was selected as the primary effect measure; for continuous outcome variables, the mean difference (MD) with its 95% CI was chosen as the effect measure. In assessing the statistical heterogeneity among the included studies, we utilized the P-value and the I2 statistic. When the P-value exceeds 0.1 and the I^2^ statistic is less than or equal to 50%, it indicates low heterogeneity among the studies, and thus, a fixed-effects model was applied for the meta-analysis. Conversely, when the P-value is less than or equal to 0.1 or the I^2^ statistic exceeds 50%, it suggests significant heterogeneity among the studies, necessitating the use of a random-effects model for analysis. In cases of considerable heterogeneity, sensitivity analysis was performed by sequentially excluding each study to test the stability of the results. Additionally, descriptive analysis was conducted to explore potential sources of heterogeneity. To further assess publication bias, funnel plots and Egger’s regression analysis were utilized. The significance level for this meta-analysis was set at α = 0.05.

## Results

### Literature Search Results

**Fig. 1:**
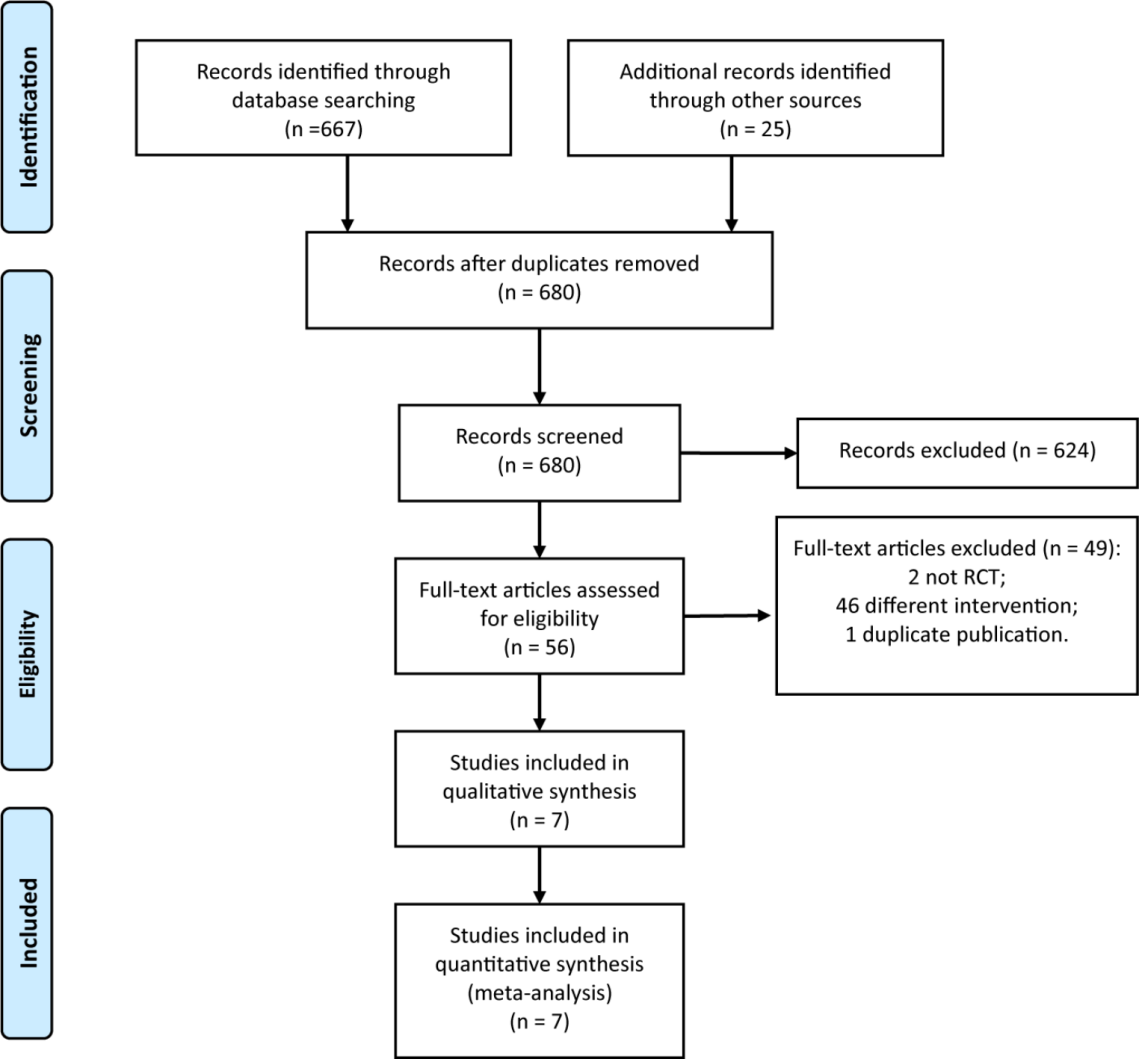
PRISMA flow diagram of study selection

The initial search yielded a total of 692 articles. After deduplication, 680 articles remained. By reviewing the titles and abstracts, 624 articles were excluded. After full-text review, an additional 49 articles were excluded, resulting in the inclusion of 7 RCTs [17–23]. The process of literature screening is depicted in Fig. 1.

### Characteristics of Included RCTs

A total of 680 patients with recurrent miscarriage were involved amongst the included 7 RCTs [17–23], 365 patients underwent G-CSF intervention, 315 patients underwent routine treatment. The characteristics of included RCTs are presented in Table 1.

**Table 1:** The characteristics of included RCTs

RCT	Country	Sample size		Methods of G-CSF administration	G-CSF dose	Duration of G-CSF intervention	Control group
		G-CSF group	Control group				
Eapen 2019 [17]	USA	76	74	Subcutaneous injection	130 ug/day	Up to 9 weeks	Placebo
Guo 2022 [18]	China	50	50	Subcutaneous injection	150 ug/ week	2 weeks	Routine treatment
Jin 2020 [19]	China	30	30	Intra-uterine perfusion	300 ug (1 mL)	Until 12 weeks of pregnancy	Routine treatment
Li 2015 [20]	China	56	44	Intra-uterine perfusion	300 ug (2 mL)	2 cycles	Normal saline
Ni 2021 [21]	China	80	40	Subcutaneous injection	1.5 U·(ug·kg)^−1^	Until 10 weeks of pregnancy	Routine treatment
Wu 2023 [22]	China	50	50	Subcutaneous injection	125 µg, every other day,	Until 8 weeks of pregnancy	Routine treatment
Zafardoust 2017 [23]	Iran	23	27	Intra-uterine perfusion	300 ug	2 cycles	Routine treatment

Granulocyte colony-stimulating factor, G-CSF

### Quality of Included RCTs

**Fig. 2:**
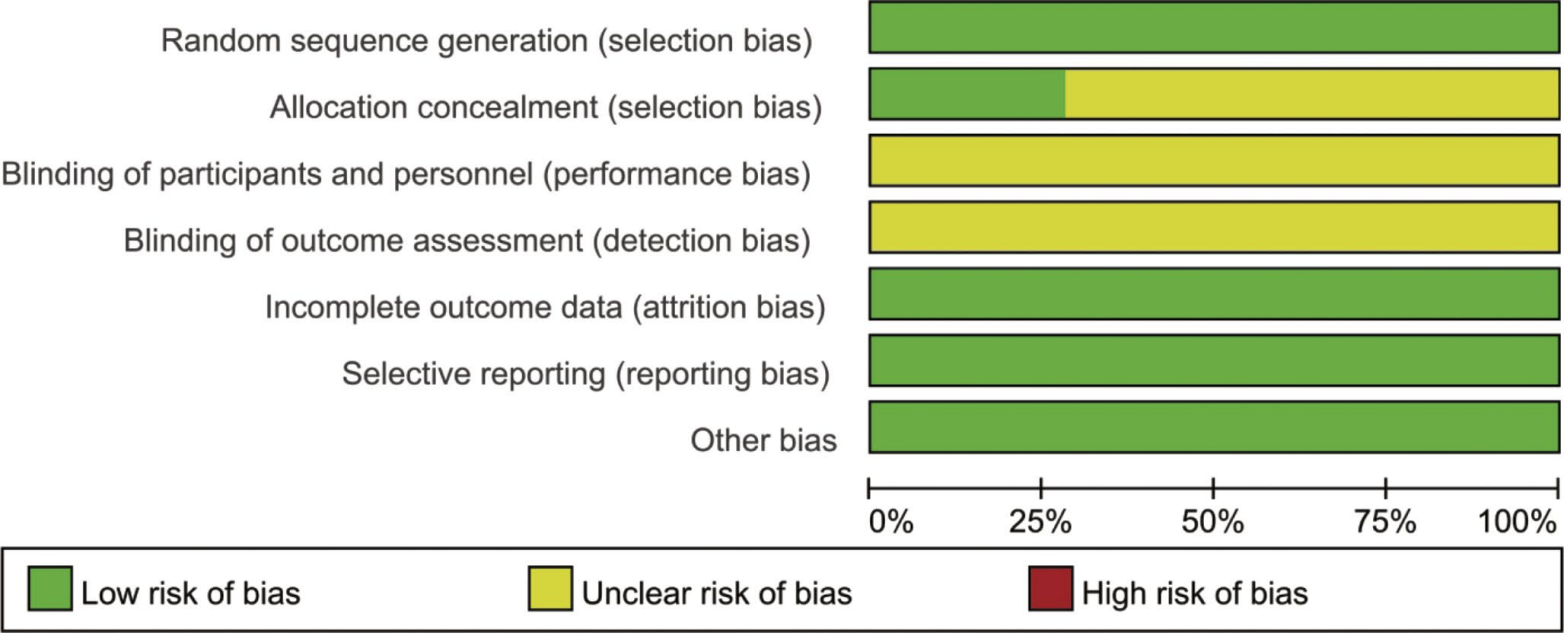
Risk of bias graph

**Fig. 3:**
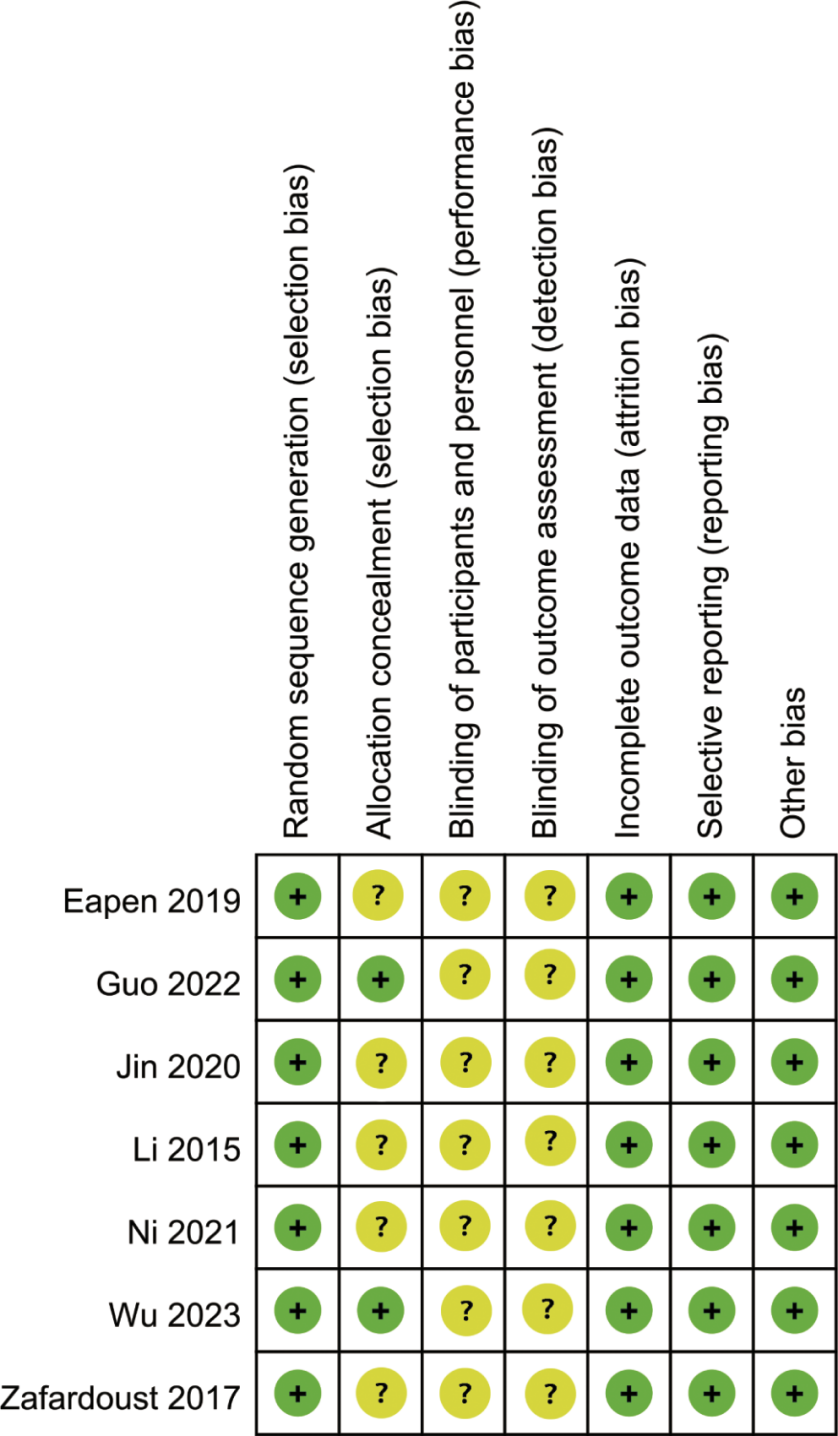
Risk of bias summary

All RCTs mentioned random grouping and provided a clear method of randomization, but only two studies reported a clear method of concealed random allocation, none of the studies used appropriate blinding methods, all studies had complete outcome data with no missing or dropout, and all studies did not selectively report results or have other biases. The results of the risk of bias assessment for the included studies are shown in Figs. 2 and 3.

### Meta-Analysis

**Fig. 4:**
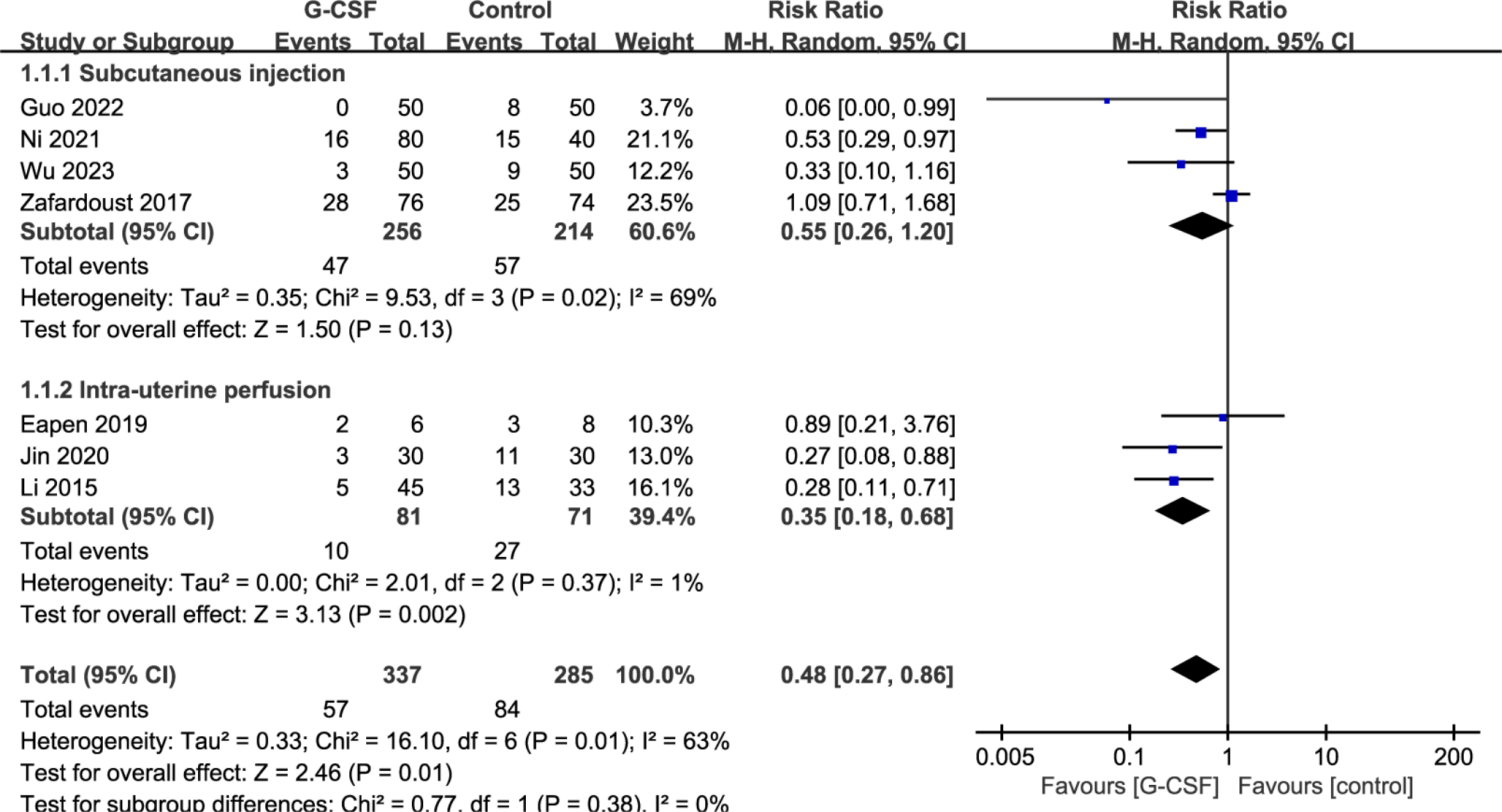
Forest plot for the abortion rate of G-CSF administration by subcutaneous injection or intra-uterine perfusion

The seven RCTs included in this analysis all reported the impact of G-CSF intervention on the abortion rate, as depicted in Fig. 4. There was significant heterogeneity among the included RCTs (I^2^ = 63%, *P* = 0.01), necessitating the use of a random-effects model for comparison. The results indicated that G-CSF intervention significantly reduced the abortion rate in patients with recurrent miscarriage [RR = 0.48, 95%CI (0.27, 0.86), *P* = 0.01]. We conducted a subgroup analysis based on the method of G-CSF administration. As shown in Fig. 4, results of subgroup analysis indicated that G-CSF administration by intra-uterine perfusion significantly reduced the abortion rate in patients with recurrent miscarriage [RR = 0.35, 95%CI (0.18, 0.68), *P* = 0.002], no statistical difference in the abortion rate was found when G-CSF was given by subcutaneous injection [RR = 0.55, 95%CI (0.26, 1.20), *P* = 0.13].

**Fig. 5:**
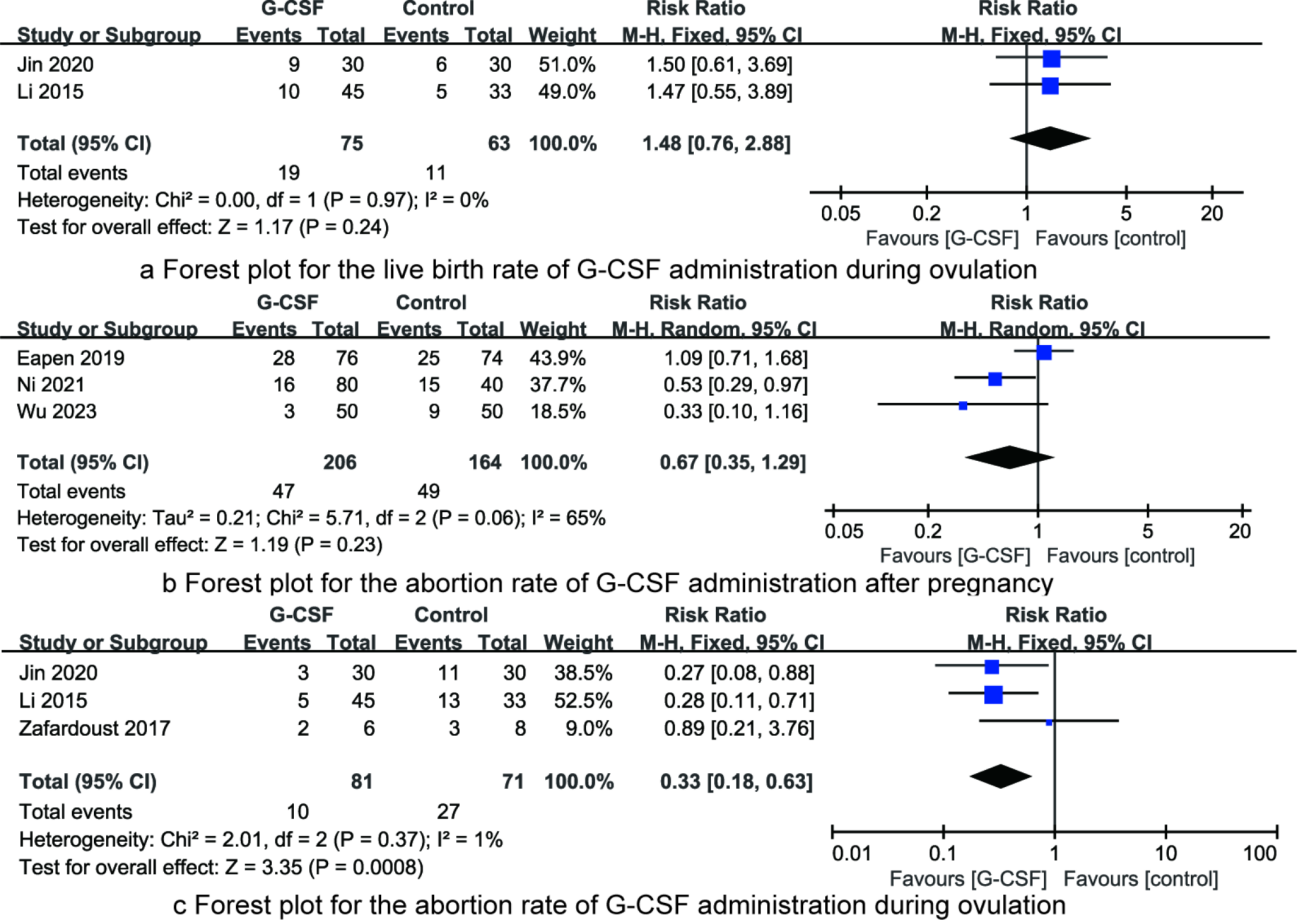
Forest plots for synthesized outcomes

Two RCTs included in this analysis reported the live birth rate of G-CSF administration during ovulation, as depicted in Fig. 5a. There was no significant heterogeneity among the included RCTs (I^2^ = 0%, *P* = 0.97), necessitating the use of a fixed-effects model for comparison. The results indicated that G-CSF intervention had no significant effect on the live birth rate in patients with recurrent miscarriage when G-CSF was administrated during ovulation [RR = 1.48, 95%CI (0.76, 2.88), *P* = 0.24].

Three RCTs included in this analysis reported the abortion rate of G-CSF administration after pregnancy, as depicted in Fig. 5b. There was significant heterogeneity among the included RCTs (I^2^ = 65%, *P* = 0.06), necessitating the use of a random-effects model for comparison. The results indicated that G-CSF intervention had no significant effect on the abortion rate in patients with recurrent miscarriage when G-CSF was administrated after pregnancy [RR = 0.67, 95%CI (0.35, 1.29), *P* = 0.23].

Three RCTs included in this analysis reported the abortion rate of G-CSF administration during ovulation, as depicted in Fig. 5c. There was no significant heterogeneity among the included RCTs (I^2^ = 1%, *P* = 0.37), necessitating the use of a fixed-effects model for comparison. The results indicated that G-CSF administration during ovulation reduced the abortion rate in patients with recurrent miscarriage [RR = 0.33, 95%CI (0.18, 0.63), *P* < 0.001].

### Publication Bias

**Fig. 6:**
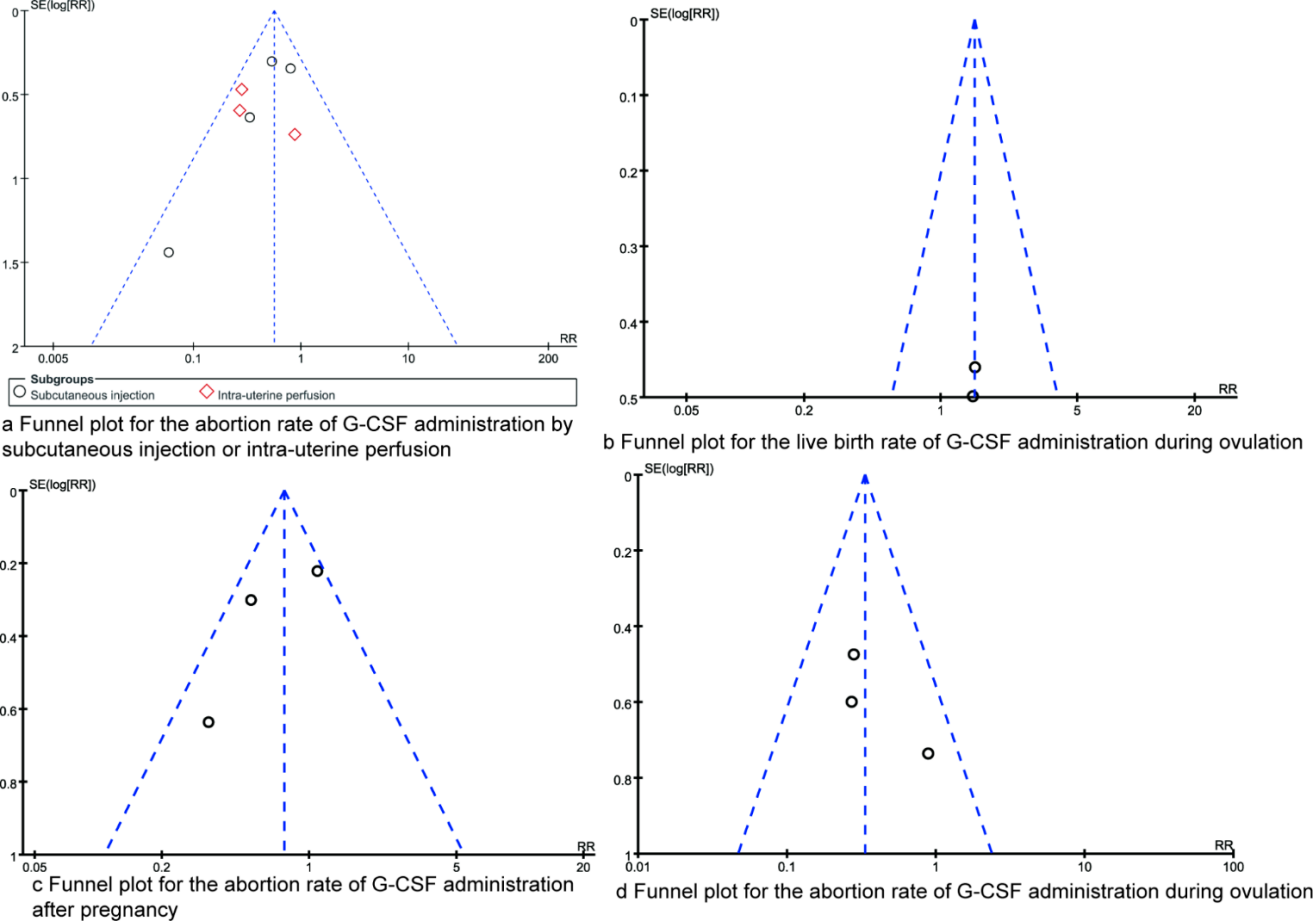
Funnel plots for synthesized outcomes

Figure 6 illustrates the inverted funnel plots for the outcomes of various meta-analyses, which exhibit a relatively symmetrical distribution of points. This pattern suggests that the likelihood of publication bias is minimal. Furthermore, the findings from the Egger regression analysis corroborate this observation, demonstrating no statistically significant publication bias across the results (all *P* > 0.05).

## Discussions

Recurrent miscarriage is a prevalent clinical pathological pregnancy condition characterized by high complexity in its prevention and treatment, and it has become a hot topic in reproductive medical research. The process of embryo implantation plays a crucial role in pregnancy, being a key determinant of pregnancy success rates [24]. The failure to conceive in some patients is closely associated with immune function abnormalities [25]. G-CSF enhances oxidative stress responses and phagocytic functions, influencing gene expression in the endometrium, and possesses physiological functions such as regulating embryonic development and promoting the revascularization of the endometrial lining [26–28]. Through paracrine mechanisms, G-CSF modulates the activation and decidualization of endometrial stromal cells, thereby facilitating embryo implantation and increasing the likelihood of a successful pregnancy [29]. However, some studies [30, 31] suggest that G-CSF does not significantly affect pregnancy or miscarriage rates. The findings of this meta-analysis indicate that intrauterine administration of G-CSF during the luteal phase and at ovulation can reduce the risk of miscarriage in patients with recurrent miscarriage and increase the live birth rate, highlighting its significant clinical application value in the treatment of recurrent miscarriage.

In the current administration methods of G-CSF, subcutaneous injection and intrauterine perfusion are the primary approaches. Reports on the impact of different administration methods on the miscarriage rate in patients with recurrent miscarriage have shown varying results. Intrauterine perfusion of G-CSF stimulates the proliferation and migration of vascular endothelial cells, enhances the anti-inflammatory effect in the basal layer of the endometrium, and stimulates neovascularization [32, 33]. This increases the perfusion of arterial blood in the basal layer, maintaining a balance of relevant cytokines in the endometrium, creating an environment more conducive to embryo implantation, and thus improving the live birth rate [34, 35]. Additionally, intrauterine perfusion helps to elevate progesterone levels, enhance the resistance to vasoactive drugs, and increase the sensitivity of the endometrium to estrogen, which is beneficial for the proliferation and hyperplasia of endometrial cells [36, 37].

The role of G-CSF in follicular development and ovulation is crucial. G-CSF is known to modulate the function of ovarian and granulosa cells, which are essential for the maturation and release of the oocyte [38]. This regulation is believed to contribute to a more favorable environment for fertilization and early embryonic development. Furthermore, G-CSF’s influence on endometrial decidualization is another key factor [39]. Decidualization is a critical process that prepares the endometrium for the implantation of the embryo. G-CSF’s involvement in this process is thought to enhance the receptivity of the endometrium, facilitating the attachment and growth of the embryo. Moreover, the study highlights the importance of G-CSF in promoting endometrial cell proliferation and embryo implantation [40]. The endometrium is a dynamic tissue that undergoes significant changes during the menstrual cycle to support pregnancy. G-CSF’s role in stimulating the proliferation of endometrial cells is crucial for creating an environment that is conducive to the implantation and growth of the embryo [41]. In addition to these direct effects, G-CSF has also been reported to have a significant impact on the maturation of trophoblasts [42]. Trophoblasts are essential cells that form the outer layer of the blastocyst and play a critical role in the implantation process [43]. The maturation of trophoblasts is crucial for the establishment of a successful pregnancy, and G-CSF’s influence on this process is thought to be a major factor in reducing miscarriage rates and increasing live birth rates [44, 45]. The timing of G-CSF administration is therefore critical. Administering G-CSF during the ovulatory phase, when the endometrium is most receptive to implantation, can maximize its beneficial effects. This precise timing can lead to a more favorable outcome for patients, potentially reducing the risk of miscarriage and increasing the chances of a successful pregnancy.

This meta-analysis has several limitations that should be considered. Firstly, the number of studies and sample size included in this research is relatively limited, which may lead to insufficient statistical power. Secondly, there are certain differences in intervention time and evaluation indicators among the studies, making it difficult to conduct further subgroup analyses due to the limited data collected. Lastly, most of the included studies are from China, which may result in some regional or population differences in the research findings. In the future, more large-sample, multi-center, high-quality RCTs are needed to verify the efficacy and safety of G-CSF treatment for recurrent miscarriage, and to further standardize the administration method, timing, and dosage of G-CSF, providing evidence-based support for the clinical treatment of recurrent miscarriage.

## Conclusion

In summary, the results of this meta-analysis indicate that intrauterine administration of G-CSF and its use during the ovulatory phase can significantly reduce the risk of miscarriage for patients with recurrent miscarriage and increase the rate of live births, making it worthy of promotion and application. The intrauterine administration of G-CSF, which involves the direct delivery of the medication into the uterine cavity, may offer a targeted approach to treatment, potentially enhancing the drug’s effectiveness while minimizing systemic side effects. Similarly, the timing of G-CSF administration during the ovulatory phase, a critical period in the menstrual cycle, suggests a strategic intervention that aligns with the natural reproductive process. The positive outcomes associated with G-CSF treatment highlight the need for continued research and clinical trials to refine treatment protocols. This includes optimizing the dosage, frequency, and duration of G-CSF administration to maximize therapeutic benefits while ensuring patient safety. The translation of these research findings into clinical practice could offer new hope to patients struggling with recurrent miscarriage, potentially improving reproductive outcomes and contributing to a better understanding of the underlying mechanisms involved in this complex condition.

## Data Availability

The data associated with the paper are not publicly available but are available from the corresponding author on reasonable request.

## Abbreviations

G: CSF-Granulocyte colony-stimulating factor
PRISMA: Preferred Reporting Items for Systematic reviews and Meta-Analyses
CNKI: China National Knowledge Infrastructure
RCTs: Randomized controlled trials
RR: Relative risk
CI: Confidence interval
MD: Mean difference

## References

[1] Jia D, Sun F, Han S, Lu L, Sun Y, Song Q. Adverse outcomes in subsequent pregnancies in women with history of recurrent spontaneous abortion: a meta-analysis. J Obstet Gynaecol Res. 2024;50(3):281–97. 38073001 10.1111/jog.15848

[2] La X, Wang W, Zhang M, Liang L. Definition and multiple factors of recurrent spontaneous abortion. Adv Exp Med Biol. 2021;1300:231–57. 33523437 10.1007/978-981-33-4187-6_11

[3] Quenby S, Gallos ID, Dhillon-Smith RK, Podesek M, Stephenson MD, Fisher J, et al. Miscarriage matters: the epidemiological, physical, psychological, and economic costs of early pregnancy loss. Lancet. 2021;397(10285):1658–67. 33915094 10.1016/S0140-6736(21)00682-6

[4] Fee N, McEvoy A, Cullen S, Doyle S, Crosby D, Allen C. Pregnancy outcomes following recurrent miscarriage. Ir J Med Sci. 2023;192(5):2255–8. 36757518 10.1007/s11845-023-03305-w

[5] Goncalves C, Feitosa BM, Cavalcante BV, Lima A, de Souza CM, Joventino LB, et al. Obesity and recurrent miscarriage: the interconnections between adipose tissue and the immune system. Am J Reprod Immunol. 2023;90(3):e13757. 37641378 10.1111/aji.13757

[6] John DG, Carlin WV. An unusual case of surgical emphysema. J Laryngol Otol. 1986;100(10):1209–11. 3772247 10.1017/s0022215100100854

[7] Bilardi JE, Temple-Smith M. We know all too well the significant psychological impact of miscarriage and recurrent miscarriage: so where is the support? Fertil Steril. 2023;120(5):937–9. 37648144 10.1016/j.fertnstert.2023.08.951

[8] He Y, Su X, Li H, Tang R, Ju Y, Chen S, et al. Subcutaneous injection granulocyte colony-stimulating factor (G-CSF) is superior to intrauterine infusion on patients with recurrent implantation failure: a systematic review and network meta-analysis. J Reprod Immunol. 2024;163:104250. 38669790 10.1016/j.jri.2024.104250

[9] Cavalcante MB, Costa Fda S, Barini R, Araujo Junior E. Granulocyte colony-stimulating factor and reproductive medicine: a review. Iran J Reprod Med. 2015;13(4):195–202. 26131007 PMC4475767

[10] Cuadrado-Torroglosa I, Garcia-Velasco JA, Alecsandru D. Maternal-fetal compatibility in recurrent pregnancy loss. J Clin Med. 2024;13(8). 10.3390/jcm13082379 PMC11051463 38673652

[11] Li N, Guan Y, Liu J, Ren B, Du Y, Wang K, et al. History of recurrent implantation failure is Associated with the incidence of adverse perinatal outcomes in Singleton Live births following frozen-thawed embryo transfer cycles. Front Endocrinol (Lausanne). 2021;12:774646. 35211088 10.3389/fendo.2021.774646 PMC8861489

[12] Gan C, Chen W, Liu Q. Progress in research on production-related mechanisms between granulocyte-macrophage colony stimulating factor and regulatory interactions to improve unexplained recurrent flow T cells. Chin J Frontier Med. 2023;15(4):60–5.

[13] Han X, Zhao X, Feng X. The regulatory mechanism of granulocyte colony stimulating factor in pregnancy and its research progress in the treatment of unexplained recurrent abortion Chinese. J Reprod Contracept. 2021;41(3):6–9.

[14] Fu J, Zhu X, Sun H. Granulocyte colony stimulating factor in the treatment of unexplained recurrent abortion: a randomized controlled trial. Chin J New Drugs Clin. 2015;34(8):602–6.

[15] Page MJ, McKenzie JE, Bossuyt PM, Boutron I, Hoffmann TC, Mulrow CD, et al. The PRISMA 2020 statement: an updated guideline for reporting systematic reviews. BMJ. 2021;372:n71. 33782057 10.1136/bmj.n71 PMC8005924

[16] Sterne JAC, Savovic J, Page MJ, Elbers RG, Blencowe NS, Boutron I, et al. RoB 2: a revised tool for assessing risk of bias in randomised trials. BMJ. 2019;366:l4898. 31462531 10.1136/bmj.l4898

[17] Eapen A, Joing M, Kwon P, Tong J, Maneta E, De Santo C, et al. Recombinant human granulocyte- colony stimulating factor in women with unexplained recurrent pregnancy losses: a randomized clinical trial. Hum Reprod. 2019;34(3):424–32. 30776296 10.1093/humrep/dey393 PMC6389865

[18] Guo X. Clinical application of recombinant human granulocyte stimulating factor in the treatment of recurrent abortion. Jiangxi Med. 2022;57(10):1550–4.

[19] Jin S, Fang J, Li S. The effect of intrauterine instillation of recombinant human granulocyte colony-stimulating factor on endometrial receptivity and pregnancy outcomes in patients with recurrent miscarriage. Shaanxi Med J. 2020;49(8):592–4.

[20] Li L, Zhao C, Feng F. Clinical study on the use of granulocyte colony-stimulating factor (G-CSF) perfusion in the treatment of recurrent abortion. Reprod Contracept. 2015;35(11):791–4.

[21] Ni S, Liu J, Li L. Clinical study of granulocyte colony stimulating factor in the treatment of recurrent abortion. Hebei Med. 2021;43(24):3766–8.

[22] Wu X, Weng A, Song Z. Effect of rhG-CSF subcutaneous injection combined with conventional treatment on pregnancy outcome in patients with recurrent abortion. Chin Foreign Med Res. 2023;21(23):126–9.

[23] Zafardoust S, Akhondi MM, Sadeghi MR, Mohammadzadeh A, Karimi A, Jouhari S, et al. Efficacy of Intrauterine Injection of Granulocyte Colony stimulating factor (G-CSF) on treatment of unexplained recurrent miscarriage: a pilot RCT study. J Reprod Infertil. 2017;18(4):379–85. 29201668 PMC5691254

[24] Ali A, Elfituri A, Doumouchtsis SK, Zini ME, Jan H, Ganapathy R, et al. Managing couples with recurrent miscarriage: a narrative review and practice recommendations. Int J Gynaecol Obstet. 2024;164(2):499–503. 37431204 10.1002/ijgo.14971

[25] Chen J, Yue J, Lu Y, Li T, Li X, Zhang JY. Recurrent miscarriage and low-titer antiphospholipid antibodies. Clin Rheumatol. 2024;43(4):1327–34. 38407714 10.1007/s10067-023-06843-x PMC10944803

[26] Hou Z, Jiang F, Yang J, Liu Y, Zha H, Yang X, et al. What is the impact of granulocyte colony-stimulating factor (G-CSF) in subcutaneous injection or intrauterine infusion and during both the fresh and frozen embryo transfer cycles on recurrent implantation failure: a systematic review and meta-analysis? Reprod Biol Endocrinol. 2021;19(1):125. 34388994 10.1186/s12958-021-00810-4 PMC8361788

[27] Arefi S, Fazeli E, Esfahani M, Borhani N, Yamini N, Hosseini A, et al. Granulocyte-colony stimulating factor may improve pregnancy outcome in patients with history of unexplained recurrent implantation failure: an RCT. Int J Reprod Biomed. 2018;16(5):299–304. 30027145 PMC6046207

[28] Yang X, Tian Y, Zheng L, Luu T, Kwak-Kim J. The Update Immune-Regulatory Role of Pro- and anti-inflammatory cytokines in recurrent pregnancy losses. Int J Mol Sci. 2022;24(1). 10.3390/ijms24010132 PMC9820098 36613575

[29] Kunicki M, Lukaszuk K, Liss J, Skowronska P, Szczyptanska J. Granulocyte colony stimulating factor treatment of resistant thin endometrium in women with frozen-thawed blastocyst transfer. Syst Biol Reprod Med. 2017;63(1):49–57. 27874292 10.1080/19396368.2016.1251505

[30] Baybordi E, Mohseni J, Mosapour P. The effect of platelet-rich plasma on the improvement of pregnancy results in repeated implantation failure: a randomized controlled trial. Int J Reprod Biomed. 2022;20(9):753–60. 36340667 10.18502/ijrm.v20i9.12065 PMC9619122

[31] Kashani L, Moini A, Esfidani T, Yamini N, Mohiti S. Effect of intrauterine granulocyte-colony stimulating factor administration on in vitro fertilization outcome in women with moderate-to-severe endometriosis: an RCT. Int J Reprod Biomed. 2021;19(8):733–40. 34568734 10.18502/ijrm.v19i8.9621 PMC8458918

[32] Torky H, El-Desouky ES, El-Baz A, Aly R, El-Taher O, Shata A, et al. Effect of Intra Uterine Granulocyte Colony stimulating factor vs. human chorionic gonadotropin at Ovum pick up day on pregnancy rate in IVF/ICSI cases with recurrent implantation failure. JBRA Assist Reprod. 2022;26(2):274–9. 34786904 10.5935/1518-0557.20210056 PMC9118974

[33] Kamath MS, Kirubakaran R, Sunkara SK. Granulocyte-colony stimulating factor administration for subfertile women undergoing assisted reproduction. Cochrane Database Syst Rev. 2020;1(1):CD013226. 31978254 10.1002/14651858.CD013226.pub2 PMC6984624

[34] Isik G, Oktem M, Guler I, Oktem E, Ozogul C, Saribas S, et al. The impact of granulocyte colony-stimulating factor (G-CSF) on thin endometrium of an animal model with rats. Gynecol Endocrinol. 2021;37(5):438–45. 32611261 10.1080/09513590.2020.1786508

[35] Dakre SM, More A, Dutta S, Ulhe SM, Choudhary N. Combination therapy with platelet-rich plasma (PRP) and granulocyte colony-stimulating factor (G-CSF) for thin endometrium: a Case Report. Cureus. 2024;16(2):e54378. 38505459 10.7759/cureus.54378 PMC10948380

[36] Ding J, Wang J, Cai X, Yin T, Zhang Y, Yang C, et al. Granulocyte colony-stimulating factor in reproductive-related disease: function, regulation and therapeutic effect. Biomed Pharmacother. 2022;150:112903. 35430390 10.1016/j.biopha.2022.112903

[37] Yang Y, Ru H, Zhang S, Wu C, Dong J, Wang X et al. The Effect of Granulocyte colony-stimulating factor on endometrial receptivity of implantation failure mouse. Reprod Sci. 2024. 10.1007/s43032-024-01527-6 38600416

[38] Su Q, Pan Z, Yin R, Li X. The value of G-CSF in women experienced at least one implantation failure: a systematic review and meta-analysis. Front Endocrinol (Lausanne). 2024;15:1370114. 38694938 10.3389/fendo.2024.1370114 PMC11061619

[39] Kushniruk N, Stastna A, Fait T, Lenertova T. Feasible influence of G-CSF on clinical pregnancy outcome in oocyte donation cycles for patients with recurrent implantation failure. Med (Kaunas). 2024;60(6). 10.3390/medicina60060966 PMC11205449 38929583

[40] Wei W, Wang N, Zhu Y, Liao M, Wang B, Du T et al. GM-CSF improves endometrial receptivity in a thin endometrium rat model by upregulating HOXA10. Mol Hum Reprod. 2023;30(1). 10.1093/molehr/gaad042 38011650

[41] Wen J, Hou B, Lin W, Guo F, Cheng M, Zheng J, et al. 3D-printed hydrogel scaffold-loaded granulocyte colony-stimulating factor sustained-release microspheres and their effect on endometrial regeneration. Biomater Sci. 2022;10(12):3346–58. 35588302 10.1039/d2bm00109h

[42] Furmento VA, Marino J, Blank VC, Cayrol MF, Cremaschi GA, Aguilar RC, et al. Granulocyte colony-stimulating factor (G-CSF) upregulates beta1 integrin and increases migration of human trophoblast swan 71 cells via PI3K and MAPK activation. Exp Cell Res. 2016;342(2):125–34. 26992288 10.1016/j.yexcr.2016.03.005 PMC5338037

[43] Furmento VA, Marino J, Blank VC, Roguin LP. The granulocyte colony-stimulating factor (G-CSF) upregulates metalloproteinase-2 and VEGF through PI3K/Akt and Erk1/2 activation in human trophoblast swan 71 cells. Placenta. 2014;35(11):937–46. 25249155 10.1016/j.placenta.2014.09.003

[44] Che JH, Zheng ZM, Li MQ, Yao X. Macrophage polarization in placenta accreta and macrophage-trophoblast interactions. Am J Reprod Immunol. 2022;88(6):e13611. 36000792 10.1111/aji.13611

[45] Gao P, Zha Y, Wei L, Zhou X, Zhu S, Zhang H, et al. G-CSF: a vehicle for communication between trophoblasts and macrophages which may cause problems in recurrent spontaneous abortion. Placenta. 2022;121:164–72. 35364512 10.1016/j.placenta.2022.03.125

